# Hallucinations in Children and Adolescents: An Updated Review and Practical Recommendations for Clinicians

**DOI:** 10.1093/schbul/sby119

**Published:** 2019-02-01

**Authors:** Kim Maijer, Mark Hayward, Charles Fernyhough, Monica E Calkins, Martin Debbané, Renaud Jardri, Ian Kelleher, Andrea Raballo, Aikaterini Rammou, James G Scott, Ann K Shinn, Laura A Steenhuis, Daniel H Wolf, Agna A Bartels-Velthuis

**Affiliations:** 1 Brain Center Rudolf Magnus, University Medical Center Utrecht, Utrecht, the Netherlands; 2 Department of Psychiatry, University Medical Center Utrecht, Utrecht, the Netherlands; 3 De Bascule, Amsterdam, the Netherlands; 4 School of Psychology, University of Sussex, Brighton, UK; 5 Research & Development Department, Sussex Partnership NHS Foundation Trust, Hove, UK; 6 Department of Psychology, Durham University, Durham, UK; 7 Department of Psychiatry, Perelman School of Medicine at the University of Pennsylvania, Philadelphia, PA, USA; 8 Developmental Clinical Psychology Research Unit, Faculty of Psychology and Educational Sciences, University of Geneva, Geneva, Switzerland; 9 Developmental NeuroImaging and Psychopathology Laboratory, Department of Psychiatry, University of Geneva School of Medicine, Geneva, Switzerland; 10 Research Department of Clinical, Educational and Health Psychology, University College London, London, UK; 11 Univ Lille, CNRS UMR-9193, SCALab (PsyCHIC Team) & CHU Lille, CURE Platform, Fontan Hospital, Lille, France; 12 Department of Psychiatry, Royal College of Surgeons in Ireland, Dublin, Ireland; 13 Division of Psychiatry, Clinical Psychology and Rehabilitation, Department of Medicine, University of Perugia, Perugia, Italy; 14 Centre for Clinical Research, Faculty of Medicine, University of Queensland, Herston, Australia; 15 Metro North Mental Health, Herston, Australia; 16 Queensland Centre for Mental Health Research, Wacol, Australia; 17 Psychotic Disorders Division, McLean Hospital, Belmont, MA; 18 Department of Psychiatry, Harvard Medical School, Boston, MA; 19 University of Groningen, Faculty of Behavioural and Social Sciences, Department of Clinical Psychology and Experimental Psychopathology, Groningen, the Netherlands; 20 University of Groningen, University Medical Center Groningen, University Center for Psychiatry, Rob Giel Research center, Groningen, the Netherlands

**Keywords:** youth, psychotic experiences, assessment, intervention, (mental) health care

## Abstract

Hallucinations in children and adolescents are now known to occur on a continuum from healthy to psychopathology-related phenomena. Although hallucinations in young populations are mostly transient, they can cause substantial distress. Despite hallucinations being widely investigated, research so far has had limited implications for clinical practice. The present article has 3 main aims: (1) to review research findings since 2014 (when the last major review of the area was published); (2) to present assessment tools validated to measure hallucinations in children and adolescents; and (3) to discuss therapeutic strategies and clinical issues. We conclude by presenting a tailored care model for clinicians and outline future challenges for research.

## General Introduction

Hallucinations (“erroneous percepts in the absence of identifiable stimuli”^1^) have been widely investigated in both adult and younger populations, and new insights continue to emerge, both from studies on hallucinations (especially auditory hallucinations) in particular, as well as from complementary research on broader psychotic experiences (PE). Though hallucinations and related PE were traditionally conceptualized as intrinsic manifestations of psychotic disorders, the increasingly accepted framework is that such phenomena reflect broader trait-like phenotypes occurring on a continuum.^2,3^ The continuum of hallucinatory experiences in children and adolescents can be interpreted as resembling the distribution found in adults, with healthy children and adolescents at one end and those experiencing more clinically significant psychopathological syndromes at the other.^1,4–9^

Superficial commonalities between hallucinations in adults and young people should not, however, be allowed to obscure significant differences that may obtain between them. One key issue is hallucination prevalence. A previous systematic review on the prevalence of PE revealed higher rates in children (17%, age 9–12 years) than in adolescents (7.5%, age 13–18 years)^10^—although a recent meta-analysis^11^ of auditory hallucinations prevalence did not find such an age-group difference (12.7% in children and 12.4% in adolescents). Furthermore, prevalence rates of auditory hallucinations in adults (5.8%) and the elderly (4.5%) were found to be significantly lower than in younger populations.^11^ This is consistent with studies showing a mostly transient course of hallucinations in children and adolescents,^9,12–14^ suggesting they are frequently self-limiting and can possibly be part of typical development.^11^

Another important issue is that hallucinations may have differing significances depending on what state of the life course they are associated with. Several studies have demonstrated that hallucinations occurring in adolescence are more indicative of psychopathology than hallucinations experienced during childhood.^5,15,16^ However, despite being less indicative, childhood hallucinations can still cause distress and impaired functioning, and may, therefore, warrant clinical attention.^5,9,17^ Unfortunately, research on hallucinations in children and adolescents has had limited impact on clinical practice, primarily due to the lack of consistent definitions, differences in assessment methods, and phenomenological complexity.^18^ For example, in their meta-analysis of auditory hallucinations prevalence, Maijer et al^11^ identified 26 study samples that had used 11 different questionnaires. Such methodological variability is likely to confound the accurate reporting of hallucinations across studies of community populations.

In 2014, Jardri and colleagues^1^ synthesized research results on childhood and adolescent hallucinations as part of the *International Consortium on Hallucination Research* (ICHR) working group and provided practical recommendations for future research. The present article has 3 main aims: (1) to outline new research that has emerged since 2014; (2) to present assessment tools used to investigate hallucinations in children and adolescents; and (3) to discuss therapeutic strategies and clinical issues.

Before presenting our review, we consider some important issues about definitions. Our working definition of “hallucination” is “a sensory experience in which a person can see, hear, smell, taste, or feel something that is not there.”

Hallucinations are reported in children as young as 5 years old, and the terms “early onset hallucinations” and “very early onset hallucinations” have used to refer to hallucinatory experiences occurring in young children.^5,19^ Although these terms might imply relevance for clinical outcomes, research findings are somewhat contradictory. For example, it is unclear what early or very early age of hallucination onset suggests about clinical course. Childhood hallucinations are reported as mostly transient^9,14^ and become increasingly associated with psychopathology during later adolescence.^5,15,16^ Additionally, adult voice-hearers without the need for care or distress have a significantly younger age of hallucination onset compared to voice-hearers with the need for care.^20^ At the same time, hallucinations present at 11 years of age was shown to increase the risk for the development of later schizophreniform disorder.^21^ So, although there might be an indication that older adolescents with the onset of hallucinations are more likely to experience or develop psychopathology, it is unclear if there is an age threshold above which the presence of hallucinations significantly increases the risk for later psychopathology. Because of the current lack of consistent nosological substantiation of the terms “very early onset” and “early onset” hallucinations, we propose to define these terms in correspondence to neurodevelopmental age categories^10,11^: “very early” (or “childhood”) for ages <13 years and “early” (or “adolescent”) for ages 13–18.

A second issue is the extent to which hallucinations are persistent. The 11-year cohort study by Bartels-Velthuis et al^13^ showed an overall 6.2% persistence rate from age 7–8 to age 18–19, with a decreasing persistence trend with increasing age (23.5% from age 7–8 to age 12–13 vs 18.2% from age 12–13 to age 18–19). Since hallucinatory experiences are by definition state phenomena (in the sense of typically unfolding over a discrete period of time), their temporal persistence is plausibly an index of psychopathological significance. Indeed, the degree of persistence of hallucinations over time may be an important predictor of transition to a clinically significant disorder.^1,22^ For example, persistence of hallucinations was shown to be associated with an increased risk for psychotic and nonpsychotic psychopathology,^13,23,24^ as well as drug abuse and suicide attempts,^24^ and need for mental health care.^25^ Nevertheless, as these studies show, hallucinations and other subthreshold positive symptoms exhibit varying trajectories, and children and adolescents with transient symptoms still have worse outcome measures and reduced quality of life at follow-up than typically developing peers.^26^ In addition, studies have measured persistence of hallucinations across a range of durations (eg, 1.6 to 8.4 years,^23^ 6 years,^25^ and 11 years^13^), and there is no clear consensus on how long hallucinations should be present for them to be considered “persistent.”

We performed a search for relevant studies published from January 2014 (the preparation date of Jardri et al’s^1^ review) until July 12, 2017 in PubMed using the following search terms: ((infant[Title/Abstract] OR infancy[Title/Abstract] OR child*[Title/Abstract] pediatric[Title/Abstract] OR paediatric[Title/Abstract] OR adolescen*[Title/Abstract]) AND hallucinat*[Title/Abstract]). The search retrieved 216 articles. Screening titles, excluding case reports and specific somatic disorder related hallucinations, resulted in 57 eligible articles. These articles were used according to relevance and scope of the present article. Also, relevant articles published after July 12, 2017 were incorporated. Although we emphasize post-2014 articles in the current article, we also, for the sake of giving the fullest picture of the current state of research, discuss their congruence (or otherwise) with earlier literature.

## Research Findings Since 2014

Since the 2014 publication,^1^ new research specifically regarding hallucinations in youth has been scarce. There is accumulating evidence suggesting an impaired global functioning of youth with auditory verbal hallucinations (AVHs), even when compared with adolescents with mental disorders (but without hallucinations).^27,28^ With regard to high and heterogenic (co)morbidity rates, a recent study in help-seeking children and adolescents with auditory hallucinations confirmed that the occurrence of hallucinations is associated with the full range of psychotic, affective, anxiety, autistic, behavioral, personality, and trauma disorders as well as cognitive impairments and parent–parent and/or parent–child interaction problems; and the majority of the sample (53%) was classified with 2 or more DSM diagnoses.^5^

### Suicidality

Accumulating evidence, from both general population and clinical samples, demonstrates a strong relationship between hallucinations in children and adolescents and comorbid suicidality with an increased risk of suicide attempts.^29,30^ Moreover, hallucinations predict incident suicidal behavior in the short term (at 3-month^31^ and 12-month^31,32^ follow-up), in the medium term (when followed from childhood into adolescence^25,33^) and in the longer term (when followed from childhood and adolescence into adulthood^24,34,35^). The relationship between hallucinations and suicidal behavior cannot be simply explained by co-occurring psychopathology. Research has demonstrated that individuals with psychopathology and hallucinations have an increased risk of suicidal behavior over and above that which would be associated with psychopathology alone.^31,36^ A number of factors may contribute to the increased risk of suicidality associated with hallucinations, including direct causation (ie, command hallucinations), but also the distress caused by hallucinatory experiences in general, as well as shared risk factors, such as mental disorders (where hallucinations and suicidality may be regarded as markers of psychopathological severity), substance use and environmental (ie, trauma and stressful life events) and psychological (ie, self-esteem and emotional regulation) factors resulting in indirect pathways.^37^

### Neuroimaging

We found only one post-2014 study specifically providing some insight in hallucinations in youth through neuroimaging studies. Amico and colleagues^38^ studied 20 young people (aged 13–16 years) with AVH and 20 controls; for the AVH group they found anomalies in functional connectivity directly and indirectly involving the Default Mode Network (DMN), the Salience Network and Central Executive Network, as well as neural networks involving both primary and secondary auditory cortical regions. These findings were in line with previous work from Jardri and colleagues,^39^ showing that hallucinatory experiences emerge from a spontaneous DMN withdrawal.

### Psychological Factors

Specific psychological models of the mechanisms involved in the onset and maintenance of hallucinations have been examined. However, evidence for these models in children and adolescents is sparse. The cognitive model for positive symptoms of psychosis by Garety and colleagues^40^ asserts that higher hallucination severity is associated with higher levels of emotional disturbance, cognitive biases, and negative life events. This has also been found in children and adolescents.^41^ Cognitive biases such as jumping to conclusions,^42^ less positive schematic beliefs about self/others,^43^ and metacognitive beliefs^44^ are all associated with hallucinations in young populations. Metacognitive beliefs^44^ were also shown to be associated with unusual perceptions in adolescents from the general population, but more studies are needed to test the metacognitive model for hallucination proneness.^45^ More recently, social cognitive mechanisms such as impairments in theory-of-mind or mentalizing have been proposed as potentially key in the emergence of hallucinations in children and adolescents.^46,47^ For example, Clemmensen et al^46^ found paranoid delusions but not hallucinations to be associated with hyper-theory-of-mind. However, Pignon and colleagues^19^ did find deficits in theory-of-mind skills in children with hallucinations. Perhaps the difference in age (and thus developmental stage) between these 2 studies can explain these different findings. The identification of psychological mechanisms that are associated with the emergence and persistence of hallucinations in children and adolescents may inform indicated preventative strategies in the future.^48^

### Social and Cultural Factors

Trauma and negative life events are known to be associated with hallucinations in a bidirectional dose–response relationship.^49–51^ Trauma is also associated with the persistence of hallucinations.^13^ However, the majority of children and adolescents experiencing maltreatment do not develop hallucinations^49^ and not every child or adolescent with hallucinations has experienced negative life events.^52^ However, these studies do suggest that children exposed to current or past traumatic event(s) are more vulnerable to the presence and potentially the persistence of voices. Moreover, discontinuation of negative life events predicts discontinuation of hallucinations,^50^ providing an opportunity for targeted intervention strategies, such as programs against bullying.

Hallucinations are typically understood differently when comparing European and African samples.^53^ However, there are sparse studies of cultural factors in hallucinations involving children and adolescents. Adriaanse et al^54^ examined the prevalence and impact of PE in a large community sample of ethnic minority and majority youth in the Netherlands. They found that minority children had a 2- to 3-fold higher prevalence of PE with high impact compared with Dutch peers. In addition, religious beliefs and/or experiences may also influence prevalence estimates. For example, one study found that moderately religious adolescents were more likely to report and develop hallucinations than nonreligious adolescents.^55^ It was speculated that religious rituals and activities could have been adopted as a method of appraisal of or coping with their hallucinations. These findings point to the need to assess ethnic and cultural context when constructing intervention strategies for young people.

### Gene and Environment Factors

There is limited evidence available from studies of genetic and environmental influences on hallucinations in children and adolescents. Zavos et al^56^ reported that out of all types of PE, heritability was lowest for hallucinations. Nevertheless, in their twin study, both extreme/frequent and milder/less frequent PE in adolescents were influenced by the same genetic and environmental factors. A genomic-wide association study from Pain et al^57^ showed that PLEs show genetic overlap with psychiatric diagnoses. The results of these 2 studies support the continuum hypothesis.

## Assessment Tools

The subjective and stigmatized nature of hallucinatory experiences and an expectation of a negative response from others may act as barriers to the disclosure of hallucinations.^58^ Furthermore, clinicians may lack confidence in talking about hallucinatory experiences.^59^ When disclosure does occur and is appropriately responded to, there are few psychometric tools for hallucinations that are suited to the cognitive and literacy levels of children and adolescents.^1^ In addition, assessment tools for hallucinations need to be directed at children and adolescents themselves, as parents are not always aware of their children’s experiences, and may report lower levels of symptoms.^9,12,60^ There are a number of instruments that more broadly assess PEs during childhood and adolescence (table 1). However, these measurements commonly assess the presence of hallucinations in the auditory domain only, using just a single item (eg “Have you ever heard voices or sounds that no one else can hear”).^19,29^ Merely assessing the presence of hallucinations may be useful, but does not incorporate specific characteristics and qualities of hallucinations. For the purpose of this review, the focus will be on outlining (new) instruments specifically designed to assess the phenomenology of hallucinations in youth.

**Table 1.** Instruments That Assess for the Presence of Hallucinations in Children and Adolescents^a^

Instrument	Authors	Properties	Validity in Children/Adolescents^b^	Hallucination Item(s)
*Instruments that comprehensively evaluate for a broad range of psychiatric disorders and/or symptoms in children and adolescents*				
CAPA	Angold et al^61^	Semi-structured diagnostic interview for ages 9–18	^b^	“Do you ever hear things that other people can’t hear? Or see things that other people can’t see? Do you ever notice smells or tastes that other people don’t?”
DISC	Shaffer et al^62^	Semi-structured diagnostic interview for ages 6–18	^b^	YCH56. “In the last year, have you ever seen something or someone that other people who were present could not see, that is had a vision when you were completely awake?” YCH57. “In the last year, have you heard things other people could not hear, such as a voice?”
K-SADS	Kaufman et al^63^	Semi-structured diagnostic interview for ages 6–18	^b^	“Has there ever been a time when you heard voices that other people could not hear? ... Did you ever hear music which other people could not?” “Has there ever been a time when you saw things like people or figures that other people could not see?” “Has there ever been a time when you smelled things that other people could not smell or felt things that were not there?”
*Instruments that screen for a broad range of psychiatric disorders and/or symptoms in children and adolescents*				
BASC	Reynolds and Kamphaus^64^	Commercially available (www.pearsonclinical.com) comprehensive screening system for measuring behavioral and emotional strengths and weaknesses in children and adolescents in preschool through high school; system consists of a teacher rating scale and a parent rating scale in addition to a 176-item self-report form; items 1–69 on self-report form are rated true (T) or false (F), while items 70–176 are rated never (N), sometimes (S), often (O), or almost always (A)	The “atypicality” scale of BASC-2, consisting of 9 items that assess symptoms similar to those identified by psychosis risk screeners (odd behaviors, delusional thoughts, paranoia, and hallucinations), was validated against the SIPS in 70 help-seeking youth ages 12–22; sensitivity 65%, specificity 87%, PPV 80%, NPV 76%^65^	62. “Sometimes, when I’m alone, I hear my name.” 122. “I hear voices in my head that no one else can hear.” 130. “I see weird things.” 160. “I hear things that others cannot hear.”
YSR	Achenbach^66^	Widely used 112-item self-report questionnaire, derived from the Child Behavior Checklist (CBCL); rated on a 3-point Likert scale (“0 = not true,” “1 = somewhat or sometimes true,” “2 = very true or often true”)	In a birth cohort of 3801 individuals born in Australia between 1981 and 1984 and enrolled in the Mater-University Study of Pregnancy and its Outcomes (MUSP), higher YSR scores at age 14 were associated with increased risk of screening positive for nonaffective psychosis (SP-NAP) on the CIDI (WHO, 1992) at age 21, in males. +AH on the YSR at age 14 showed a 5.1-fold odds (95%	40. “I hear sounds or voices that other people think aren’t there.” 70. “I see things that other people think aren’t there.”
			CI: 2.2–11.8) in males and a 2.3-fold odds (95% CI: 1.0–5.1) in females of becoming SP-NAP at 21. +VH on the YSR at age 14 was associated with a 2.9-fold odds (95% CI: 1.1–7.5) of becoming SP-NAP at 21.^67^ Individuals from the MUSP birth cohort study (250 who reported YSR hallucinations only at age 14, 83 who reported YSR hallucinations at both 14 and 21 years, and 321 who did not endorse hallucinations at either 14 or 21) were assessed again at age 30–33; hallucinations at age 14 alone not associated with any mental disorder in adulthood vs hallucinations endorsed at both 14 and 21 years had increased odds of being diagnosed with a psychotic disorder, a substance use disorder, and lifetime suicide attempts.^24^ The “thought problems” subscale of the YSR (9 items, including AH and VH) was also used to prospectively track different trajectories of subclinical psychotic experiences among adolescents in the general Dutch population participating in the TRacking Adolescents’ Individual Lives Survey (TRAILS).^25^	
*Instruments that comprehensively evaluate for psychosis or psychosis-like experiences in children and adolescents*				
CAARMS	Yung et al^68^	Comprehensive diagnostic interview and rating system to assess psychosis risk	^b^	“Do you have visions, or see things that may not really be there? Do you ever see things that others can’t, or don’t seem to?...” “Do you ever hear things that may not really be there? Do you ever hear things that other people seem not to (such as sounds or voices)?...” “Do you ever smell things that other people don’t notice?...” “Do you ever get any odd tastes in your mouth?...” “Do you ever get strange feelings on, or just beneath, your skin?...” “Have you noticed any change in your bodily sensations, such as increased, or reduced intensity? Or unusual bodily sensations such as pulling feelings, aches, burning, numbness, vibrations?”
SIPS	Miller et al^69^	Structured interview to diagnose the psychosis prodrome; consists of the Scale of Prodromal Symptoms (SOPS), Schizotypal Personality Disorder Checklist, family history questionnaire, and global assessment of functioning (GAF)	^b^	*Auditory:* 3. “Do you ever hear unusual sounds like banging, clicking, hissing, clapping, or ringing in your ears?” 4. “Do you ever think you hear sounds and then realize that there is probably nothing there?” 5. “Do you ever hear your own thoughts as if they are being spoken outside your head?” 6. “Do you ever hear a voice that others don’t seem to or can’t hear?” *Visual:* 3. “Have you ever seen unusual things like flashes, flames, vague figures or shadows out of the corner of your eye?” 4. “Do you ever think you see people, animals, or things, but then realize they may not really be there?” 5. “Do you ever see things that others can’t or don’t seem to see?” *Somatic:* 1. “Have you noticed any unusual bodily sensations such as tingling, pulling, pressure, aches, burning, cold, numbness, vibrations, electricity, or pain?” *Olfactory and gustatory:* 1. “Do you ever small or taste things that other people don’t notice?”
*Instruments that screen for psychosis or psychosis-like experiences in children and adolescents*				
APSS	Kelleher et al^70^	7-item self-report questionnaire (4 items from DISC^2^ plus additional questions on visual hallucinations, delusions of control, and grandiosity); includes 3-choice response (“Yes, definitely” = 1 point, “Maybe” = 0.5 point, “No, never” = 0 point).	Validated against the K-SADS in 334 adolescents ages 11–13 in the school setting in Dublin, Ireland; PPV 100% and NPV 88.4% for any psychotic-like experiences (PPV 71.4%, NPV 90.4% for AH).^70^	4. “Have you ever heard voices or sounds that no one else can hear?” 6. “Have you ever seen things that others cannot see?”
CAPE-42	Stefanis et al^71^	42-item self-report questionnaire modified from PDI-16^72^; includes 4-choice response (“never,” “sometimes,” “often,” “nearly always”); for any response other than “never,” respondent is instructed to indicate degree of distress caused by the experience	Validated against the CAARMS in 165 help-seeking youth ages 13–24; PPV 65%, NPV 63% for full questionnaire (cutoff of 3.2 in the positive dimension subscale showed sensitivity 67%, specificity 73%, PPV 72%, NPV 68%; cutoff of 2.8 showed sensitivity 83%, specificity 49%, PPV 63%, NPV 74%)^73^	30. “Do you ever hear your own thoughts being echoed back to you?” 33. “Do you ever hear voices when you are alone?” 34. “Do you ever hear voices talking to each other when you are alone?” 42. “Do you ever see objects, people, or animals that other people cannot see?”
DAWBA-PE-S	Gundersen et al^74^	10 self-report items on psychotic experiences (PE), or “strange experiences that are surprisingly common”; PE section is embedded within the DAWBA, a comprehensive online questionnaire administered to parents, children (ages 11+), and teachers (PE items answered only by the child); rated on a 3-point Likert scale (“0 = no,” “1 = a little,” “2 = a lot”).	Validated against 22 K-SADS psychosis items in 1571 children ages 11–12 participating in a longitudinal birth cohort study in Copenhagen, Denmark (Copenhagen Child Cohort 2000); sensitivity 74%, specificity 77%, PPV 27%, NPV 96%^74^	T1. “Anthony sees visions. He sees people, animals or other things that seem unreal to him but that can’t be seen by other people even if they are there at the time. Do you ever see visions?” T2. “Bill hears special voices inside his head. For example, he hears strange voices talking to him or about him. Do you ever hear special voices inside your head?” T3. “Charles hears special voices coming out of the air when there is definitely no one around. This is not just him imagining that he has heard someone calling his name (which is extremely common). He hears much more than this: conversations about himself or people talking a lot to him. Do you ever hear special voices from outside yourself?”
HQ^c^	Posey and Losch^75^; Pearson et al^76^	14-item self-report questionnaire (version modified for adolescents by Pearson et al^76^ contains 12 items—voice of God and driving-related items excluded); binary response (yes/no)	Modified version was used to assess hallucinatory experiences in a nonclinical sample of 250 adolescents in the UK ages 14–15 (compared with a nonclinical adult sample)^76^	1. “Sometimes I have thought I heard people say my name…like in a store when you walk past some people you don’t know … Has something like this ever happened to you?” 5. “When I was little, I had an imaginary playmate, I remember that I really thought I heard her voice when we talked… a) Did you have an imaginary playmate? b) Did you hear his/her voice aloud?” 6. “Every now and then—not real often—I think I hear my name on the radio. Happened to you?” 7. “Sometimes when I’m in the house all alone, I hear a voice call my name … I guess I kind of know that it really isn’t somebody and it’s really me … but it does sound like a real voice. Happened to you?” 8. “Last summer I was hanging up clothes in the backyard. Suddenly I heard my [husband] call my name from inside the house. He sounded like something was wrong and was loud and clear. I ran in … but he was out in the garage and hadn’t called at all…. This or something similar happen to you?” 9. “I’ve heard the doorbell or the phone ring when it didn’t. Happen to you?” 10. “I hear my thoughts aloud. Happen to you?” 11. “I have heard God’s voice … not that he made me know in my heart … but as a real voice. Happen to you?”
				12. “When I am driving in my car … particularly when I’m tired or worried … I hear my own voice from the backseat … usually soothing … Similar things happen to you?” 13. “I drive a lot at night…. Sometimes late at night, when I’m tired, I hear sounds in the backseat like people talking … just a word here and there … Anything similar happen to you?” 14. “Almost every morning … I have a pleasant conversation with my dead grandmother. I talk to her and quite regularly hear her voice actually aloud. Anything similar happen to you?”
LHSH-R	Launay and Slade^77^; Bentall and Slade^78^	12-item self-report questionnaire; revised version by Bentall and Slade^78^ rated on a 5-point Likert scale (“0 = certainly does not apply to you,” “1 = possibly does not apply to you,” “2 = you’re unsure,” “3 = possibly applies to you,” “4 = certainly applies to you”).	Items #7 and 12 were used to assess for AVH in a population-based sample of 9646 Norwegian adolescents ages 16–19^79^	2. “In my daydreams I can hear the sound of a tune almost as clearly as if I were actually listening to it.” 5. “The sounds I hear in my daydreams are usually clear and distinct.” 7. “I often hear a voice speaking my thoughts aloud.” 8. “In the past I have had the experience of hearing a person’s voice and then found that no one was there.” 9. “On occasions I have seen a person’s face in front of me when no one was in fact there.” 10. “I have heard the voice of the devil.” 11. “In the past I have heard the voice of God speaking to me.” 12. “I have been troubled by hearing voices in my head.”
PLEQ-C	Laurens et al^80^	9-item self-report questionnaire (5 items adapted from DISC^2^ plus 4 additional items); includes 3-choice response (“0 = not true,” “1 = somewhat true,” “2 = certainly true”)	Factor analytic methods were used to determine the latent structure of psychosis-like experiences in 7966 children ages 9–11 recruited from 73 primary schools in the greater London area; the 2 hallucination items were most able to discriminate a latent psychotic-like construct from dimensions representing internalizing and externalizing problems^80^	4. “Have you ever heard voices that other people could not hear?” 9. “Have you ever seen something or someone that other people could not see?”
PLIKSi	Horwood et al^81^	Semi-structured interview with 12 “core” items (7 items from DISC^2^ + 5 items from SCAN^82^) covering 3 domains of positive psychotic symptoms (hallucinations, delusions, bizarre symptoms); includes 3-choice response [“yes” (Y), “no” (N), “maybe” (M)] and additional options for “refused” (R) and “don’t know” (DK); if child answers “yes” or “maybe,” interviewer uses supplementary questions to probe (eg, items H2–H11, H18–H19). Interviewer is also prompted to ask if the specific item in question only ever happened when falling asleep or waking up, when ill with a high temperature, when drinking alcohol, or while using drugs (cannabis, amphetamines/speed, glue, others, or not applicable).	In 6455 children, mean age 12.9 years (range 11.4–14.3), from the Avon Longitudinal Study of Parents and Children (ALSPAC) birth cohort in the UK, PPV’s when comparing self-report with the final interviewer ratings, were poor (3%–50%) for all items except AH (71%)^81^	Auditory: H1. “Since your 12th birthday have you ever heard voices that other people can’t hear?” H2. “What did you hear? What kinds of things did you hear? What did you think it was? Did you think it was your imagination or real? Was it like a thought or more like a voice?” H3. “How often have these voices happened to you since your 12th birthday?” H4. “The voices that you have heard, where did they come from? From inside your head? Was it your thoughts you heard? Could other people hear the voices?” H5. “From outside your head, through your ears? Did it sound as clear as my voice does talking to you right now?” H6. “Do the voices talk directly to you or tell you things?” H7. “Do these voices tell you anything? (what?) (Good or bad?) Have they ever told you to hurt yourself or kill yourself? Have they ever told you to hurt or kill someone else? Who? How?” H8. “Do you hear voices that talk about what you’re doing? Or feeling? Or thinking?” H9. “Do you ever hear 2 or more voices (that others couldn’t hear) talking to each other? Or about you? H10. “Have there been other noises or voices you have heard that you have not told me about? [Rate here sporadic (single word) hallucinations.]” H11. “Or elementary hallucinations (noises such as bangs or bells)?” *Visual:*H17. “Since your 12th birthday have you ever seen something or someone that other people couldn’t see?” H18. “Did it see real? Can you give me an example?” H19. “How often has this occurred since your 12th birthday?”
		
			
PRIME-R	Miller et al^83^	12-item self-report questionnaire developed by the authors of the SIPS; asks about experiences within the year; rated on a 7-point Likert scale (“0 = definitely disagree,” “1 = somewhat disagree,” “2 = slightly disagree,” “3 = not sure,” “4 = slightly agree,” “5 = somewhat agree,” “6 = definitely agree”)	Validity of Japanese version tested in 528 psychiatric outpatients ages 16–30; in the 115 patients who completed both PRIME-R and SIPS, sensitivity was 100%, specificity 74%, PPV 43%, NPV 100%^84^ Validity also tested using the SIPS in 49 individuals ages 12–22 accessing mental health services in the US; using a cutoff of ≥2, sensitivity 80%, specificity 48%, PPV 52%, NPV 78%^85^ A culturally modified (mPRIME) version showed less robust validity measures in a nonclinical sample of 2758 Kenyan youth ages 14–29; in the 182 individuals who completed both the mPRIME and SIPS, sensitivity was 40%, specificity 65%, PPV 12%, NPV 90%^86^	10. “I have had the experience of hearing faint or clear sounds of people or a person mumbling or talking when there is no one near me.” 11. “I think that I may hear my own thoughts being said out loud.”
PQ-B	Loewy et al^87^	21-item (brief version) self-report questionnaire; asks about experiences within the past month; binary response (yes/no); if answer is “yes,” respondent is asked to indicate how problematic the experience is	Validated against the SIPS in 141 individuals ages 12–35 referred to 1 of 2 prodromal research clinics in the US (California); cutoff of ≥3 demonstrated sensitivity 89%, specificity 58%, PPV 93%, NPV 46%^88^ Validity also tested using the SIPS as gold standard in 49 individuals ages 12–22 accessing mental health services in the US (Maryland); cutoff of ≥6 showed sensitivity 95%, specificity 28%, PPV 48%, NPV 89%^85^	2. “Have you heard unusual sounds like banging, clicking, hissing, clapping or ringing in your ears?” 9. “Do you sometimes get strange feelings on or just beneath your skin, like bugs crawling?” 17. “Are your thoughts sometimes so strong that you can almost hear them?” 19. “Have you seen unusual things likes flashes, flames, blinding light, or geometric forms?” 20. “Have you seen things that other people can’t see or don’t seem to see?”
PQ-16	Ising et al^89^	16-item self-report questionnaire; binary response (true/false); if answer is “true,” respondent is asked to indicate how much distress he/she experiences on a 4-point scale (“0 = No,” “1 = Mild,” “2 = Moderate,” “3 = Severe”)	Validated in 3671 help-seeking Dutch adults ages 18–35^89^; Italian version (iPQ-16) validated against the CAARMS in 72 help-seeking adolescents ages 13–17 referred to child and adolescent neuropsychiatry services; sensitivity 77%, specificity 54%, PPV 72%, NPV 60%^90^	3. “I sometimes smell or taste things that other people can’t smell or taste.” 4. “I often hear unusual sounds like banging, clicking, hissing, clapping, or ringing in my ears.” 6. “When I look at a person, or look at myself in a mirror, I have seen the face change right before my eyes.” 8. “I have seen things that other people apparently can’t see.” 9. “My thoughts are sometimes so strong that I can almost hear them.” 13. “I have heard things other people can’t hear like voices of people whispering or talking.”
SPEQ	Ronald et al^91^	63-item self- and parent-report questionnaire comprised of 6 psychosis subscales; the 9 items in the hallucination subscale were selected from the CAPS^92^ and rated on a 6-point Likert scale (“0 = not at all,” “1 = rarely,” “2 = once a month,” “3 = once a week,” “4 = several times a week,” “5 = daily”)	Validity assessed via agreement with the PLIKSi in approx. 5000 16-year-old twins born in the UK (and their parents); correlation between SPEQ and PLIKSi for hallucinations was *r* = .60, *P* < .001^93^	“Hear sounds or music that people near you don’t hear?” “See things that other people cannot?” “Feel that someone is touching you, but when you look nobody is there?” “Hear noises or sounds when there is nothing about to explain them?” “Detect smells which don’t seem to come from your surroundings?” “See shapes, lights, or colors even though there is nothing really there?” “Notice smells or odors that people next to you seem unaware of?” “Experience unusual burning sensations or other strange feelings in or on your body that can’t be explained?” “Hear voices commenting on what you’re thinking or doing?”
Y-PARQ-B	Ord et al^93^	28-item (brief version) self-report questionnaire, based on the CAARMS; includes 3-choice response (“Y” = yes, “N” = no, “U” = undecided)	Validated against the SIPS in 49 individuals ages 12–22 accessing mental health services in the US; using a cutoff of ≥11, sensitivity was 65%, specificity 76%, PPV 65%, NPV 76%^85^	12. “Do you ever hear the voice of someone talking that other people cannot hear?” 15. “Have you noticed any unusual bodily sensations such as tingling, pulling, pressure, burning, cold, vibrations, drilling, tearing, or electricity?” 19. “Do you ever hear sounds that are not there?” 22. “Do you see things that others can’t or don’t see?” 24. “Do you get strange feelings on or just beneath your skin?”

*Note*: AH, auditory hallucinations; AVH, auditory verbal hallucinations; APSS, Adolescent Psychotic Symptom Screener; BASC, Behavior Assessment System for Children; CAARMS, Comprehensive Assessment of the At-Risk Mental State; CAPA, Child and Adolescent Psychiatric Assessment; CAPE, Community Assessment of Psychic Experiences; CAPS, Cardiff Anomalous Perceptions Scale; CIDI, Composite International Diagnostic Interview; DAWBA-PE-S, The Development and Well Being Assessment, Self-Reported Psychotic Experiences; DISC, Diagnostic Interview Schedule for Children; K-SADS, Kiddie Schedule for Affective Disorders and Schizophrenia for School Aged Children; HQ, Hallucination Questionnaire; LHSH-R, Launay-Slade Hallucination Scale, Revised; NPV, negative predictive power; PDI-16, Peters Delusions Inventory, 16 Item; PPV, positive predictive value; PLEQ-C, Psychotic-Like Experiences Questionnaire for Children; PLIKSi, Interview for Psychosis-Like Symptoms; PQ-16, Prodromal Questionnaire, 16 Item; PQ-B, Prodromal Questionnaire, Brief; PRIME-R, PRIME Screen, Revised; SCAN, Schedules for Clinical Assessment in Neuropsychiatry; SIPS, Structured Interview for Psychosis Risk Syndromes; SPEQ, Specific Psychotic Experiences Questionnaire; Y-PARQ-B, Youth Psychosis At-Risk Questionnaire, Brief; YSR, Youth Self Report.

^a^Only instruments available in English are listed.

^b^Validation measures in children and adolescents are described only for screening instruments.

^c^Hypnagogic and hypnopompic hallucination items in the Hallucination Questionnaire (items 2–4) not listed.

Since the 2014 article,^1^ 2 themes relating to the assessment of hallucinations have emerged. First, the introduction of developmentally appropriate methods of assessment that are suitable and comfortable for a younger generation. Second, the use of a structured, systematic and comprehensive approach to the assessment of hallucinations in youth. See table 2 for an overview of the most recently developed instruments, in line with these themes.

**Table 2.** Instruments That Assess for Phenomenological Features of Hallucinations in Youth

Instrument	Age Group	Modalities	Benefits	Drawbacks
MHASC	Very early and early onset	5 sensory modalities explored + cross-modal experiences (auditory, visual, somatosensory, gustatory, olfactory)	Attractive layout (game-based app) Systematic and comprehensive	Validation ongoing
SOCRATES	Very early and early onset	1 sensory modality explored (auditory)	Systematic and comprehensive	Requires clinical skills and presence of interviewer Not validated
AVHRS-Q	Early onset	1 sensory modality explored (auditory)	Quick assessment Systematic and comprehensive Validated	Not suitable for very early onset hallucinations

*Note*: MHASC, Multisensory Hallucinations Scale for Children; AVHRS-Q, Auditory Vocal Hallucination Rating Scale Questionnaire; SOCRATES, Assessment of Perceptual Abnormalities and Unusual Thought Content.

Regarding theme 1, it is important to acknowledge that the current generation of children and adolescents are increasingly using digital platforms. The MHASC^94^ (Multisensory Hallucinations Scale for Children) was specifically developed with this in mind and assesses quantitative and phenomenological features of hallucinations in all modalities. This app utilizes common game-based aesthetics and codes to increase engagement and motivation of children and adolescents during the assessment, using a simple, intuitive, and playful interface with developmentally appropriate language.^95^ The MHASC app was designed for use in community populations of children and adolescents. Recognizing that potential harm can come from labeling the experienced phenomena as mental illness, the developers emphasize that MHASC is not a diagnostic tool but more a quantitative and phenomenological measure. The MHASC app is currently being validated and will be made freely available on common App Stores in multiple languages.

The SOCRATES assessment of perceptual abnormalities and unusual thought content, similarly, provides a structured and comprehensive approach to assess specific characteristics of hallucinations (auditory and others), for use in children and adolescents in both clinical and research environments.^96^ It has been developed with the aim of providing a method that is standardized, systematic, and comprehensive, facilitating the assessment of changes over time and the comparison of phenomena across studies and centers.

For the assessment of specific characteristics and severity of AVH in pediatric populations, the Auditory Vocal Hallucination Rating Scale^97,98^ (AVHRS) can be used.

In adolescent (and adult) populations (aged from 12 years), the AVHRS-Q(uestionnaire) has been developed as a self-report version of the AVHRS.^99^ The AVHRS-Q can be delivered online, providing a brief (on average taking 6 min) and comprehensive assessment of AVH. The AVHRS-Q has been used in the second follow-up of a large Dutch cohort study on AVHs in 18- to 19-year-old adolescents,^13^ and is currently being validated.

To conclude, the MHASC, the SOCRATES, and the AVHRS-Q assessment are all suitable for the assessment of characteristics and phenomenology of AVH in youth and are outlined in table 2. All of these instruments are primarily developed for research purposes, although they can also be applied in clinical practice to help clinicians to standardize their assessment of hallucinations. New digital technologies and other methods have presented new opportunities for both research and clinical practice, to better reach, study, assess and if necessary treat children and adolescents with hallucinations.

## Therapeutic Strategies and Clinical Issues

Children and adolescents may seek help for hallucinations, presenting themselves to community health services, general practitioners, outpatient clinics and emergency services,^5,100–102^ and a number of treatment options are available.

### Targeted Psychoeducation and Psychotherapy

Kapur and colleagues^101^ investigated the experience of children and adolescents with hallucinations and their parents when engaging in mental health services. These young voice hearers reported feeling lost, not listened to, and found it difficult to obtain useful information. Parents sought a holistic approach (including counseling, peer groups, meditation, drug information sharing, and alternative educational opportunities), whereas the children and adolescents preferred a more normalizing and destigmatizing approach. In line with the need for a more holistic approach, psychological interventions with a transdiagnostic and symptom-specific focus are deemed more acceptable by both clinicians and children and adolescents.^101,103^ Also, Jardri and colleagues^1^ emphasized an urgent need for psychotherapeutic interventions specifically developed for children and adolescents. Furthermore, Ruffell and colleagues^104^ conclude that targeted cognitive behavioral therapy (CBT) for PE in children and adolescents is recommended to improve clinical outcome. Currently, such tailored interventions are being developed. In the UK, hallucinations are one target of the Coping with Unusual ExperienceS for children (age < 12 years) (CUES) (ISRCTN 13766770) and Coping with Unusual ExperienceS for 12–18 year olds (CUES+)^105^ (ISRCTN 21802136) studies that are evaluating CBT-informed interventions for children and adolescents with PE. In the Netherlands, Maijer and colleagues^5^ (see their supplementary material for more information) developed Stronger Than Your Voices (STYV), which is a form of CBT developed at an outpatient clinic for children and adolescents suffering from hallucinations that can be applied regardless of age or possible underlying (psychiatric) disorder. STYV is currently being assessed within a feasibility study.

The relation-based therapies for hallucinations that are being developed for adults might also be useful for young people (eg, relating therapy^106^), given their emphasis on responding in more adaptive ways to difficult relationships (irrespective of the seen [social] or unseen [auditory hallucination] nature of the relational other). This focus on relationships addresses the aforementioned need for therapy to incorporate holistic and normalizing approaches.

### Medication

Medication does not play a primary role in the treatment of hallucinations. When hallucinations are present in children and adolescents in the context of an established psychotic disorder, treatment with antipsychotic medication can be considered, following treatment guidelines.^107^ However, the presence of distressing hallucinations does not always justify the diagnosis of a psychotic disorder and thus warrants restraint in prescribing antipsychotic medication.^5,108^ Incidentally, for example, when hallucinations are a symptom or signal of decompensation of underlying conditions (such as an autism spectrum disorder or borderline personality disorder), antipsychotic medication can be considered as a (temporary and supplementary) intervention, according to related (inter)national guidelines and treatment protocols.

### Other Interventions

Other hallucination-focused interventions for children and adolescents include repeated transcranial magnetic stimulation (rTMS) as an add-on to therapy for persistent hallucinations. Although no new research on rTMS specifically for hallucinations in children and adolescents has emerged since 2014,^1^ earlier findings highlight the potential beneficial effects of low-frequency rTMS on reducing early-onset treatment-resistant hallucinations. There remains a need for large controlled trials to test its efficacy, which may aid in determining optimized stimulation parameters and evaluate its long-term therapeutic effect.

The use of virtual reality and avatars in the treatment of several dimensions of psychotic symptoms is promising,^109,110^ although there is still limited research, which is only restricted to adults at this time. In addition to digital assessment tools, online and virtual treatment strategies might be specifically appealing to children and adolescents and should be explored in future research.

### Clinical Application

When screening for hallucinations, it is important to note the potential barriers to disclosure^58^ and the possibility that parents may not always be aware of the presence and/or significance of children’s hallucinations.^9,12,60^ Moreover, children from young ages have the capacity to report on their hallucinatory experiences^5,9,11,19^ and the age-appropriate questionnaires (albeit mostly regarding auditory hallucinations) described above can be used to facilitate these conversations in both community and clinical settings.

Attention has been drawn to the needs of children and adolescents actually seeking help for hallucinations, as the duration and severity of their complaints are often substantial, and there can be a variety of (severe) comorbid psychopathology.^5^ Help-seeking children and adolescents and their parents are in need of information and targeted help to address hallucinations, preferably through a holistic and de-stigmatizing approach.^5,101^ The rise of easily accessible community services (such as Headspace in Australia, Heads Together in the United Kingdom, and @Ease in the Netherlands) might support such an approach and perhaps diminish the gap between the duration of complaints and referral to appropriate care. These community services could fulfill the need for easily accessible ways to gain information and/or to screen whether there is an indication for referring to mental health care facilities.

**Fig. 1.**
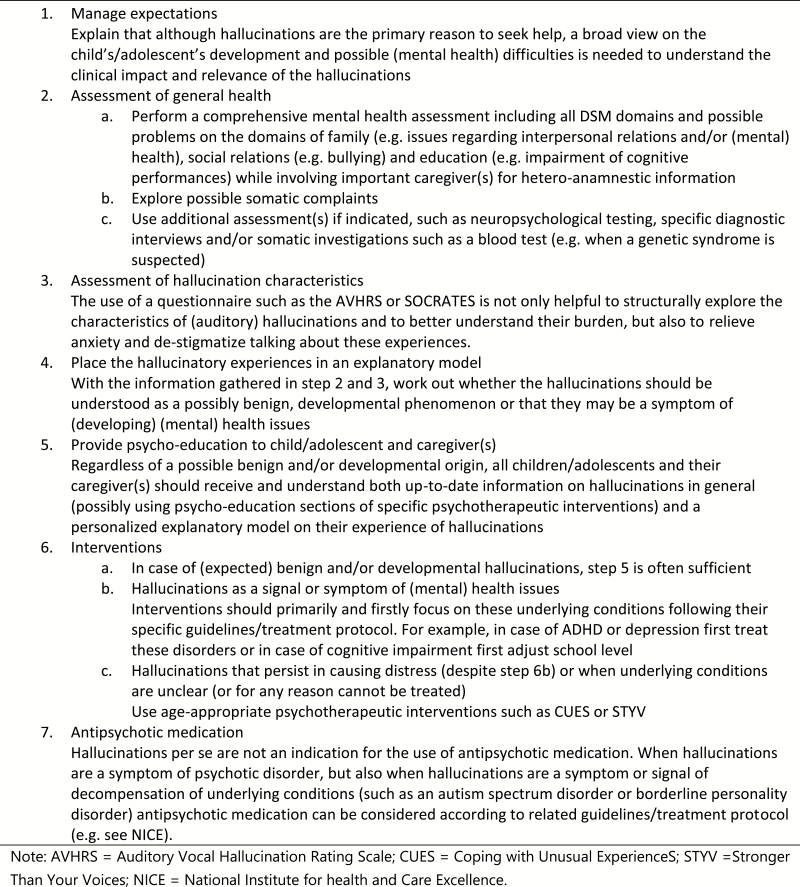
Guide for clinicians: a stepwise approach to hallucinations in youth.

When encountering children and adolescents seeking help for hallucinations, clinicians should initially adopt a “curious-but-cautious” attitude, seeking to learn more about the hallucinatory experience and its psychosocial and psychopathological context. A holistic perspective can maximize engagement at this stage and the provision of psychoeducation about hallucinations, especially within the framework of the continuum model, may be helpful. If the clinician identifies that hallucinations are present, underlying causative factors and/or (psycho)pathology should first be targeted before considering hallucination-specific interventions. The experience of the clinicians working at the specialized outpatient clinic for youth with AVH at the UMC Utrecht suggests that in many cases, hallucinations subsequently decrease or even diminish when underlying causative factors (such as psychiatric [co]morbidity) can be adequately targeted.^5^ However, if (still) indicated and/or requested, the age-appropriate psychotherapeutic interventions described above are being developed to target the hallucinations. To provide knowledge and information on hallucinations, the psychoeducation section of such treatment protocols might be used for children and their parents before (or without) applying a whole treatment protocol. When hallucinations are present in the context of a psychotic disorder and/or previous steps were not sufficient, antipsychotic medication can be considered. A stepwise guide for clinicians is given in figure 1. In addition, as hallucinations are strongly associated with suicidal behavior, it is critical to perform suicide risk assessment in young people reporting hallucinations.

## Direction for Future Research

We implicitly adopted the mainstream definition of a hallucination, as “an erroneous percept in the absence of identifiable stimuli.”^1^ However, to better understand hallucinations in children and adolescents, it seems essential to reach a more specific consensus on how to conceptualize and define hallucinatory experiences. For example, does this definition include auditory illusions and forms of inner speech (often a silent monologue without intrinsic sound or vocal quality)? Is hearing noise or music as much a hallucinatory experience as hearing voices? Are “sonorized thoughts” (thoughts with pathologically enhanced acoustic qualities) part of the hallucinatory spectrum?^111,112^ Similarly, a critical point is the consensus on suitable assessment tools (ie, consensus on the consequent use of one or more questions/questionnaires) for hallucinations, which presupposes some preliminary agreement on their core phenomenal features. Research in both youth and adult populations might benefit from stepping away from the use of single-item screening to identify individuals with hallucinations that are then regarded as a “homogeneous” study population, and rather implement the exploration of the hallucinatory experience (not only in terms of duration, distress, and frequency, but also in terms of being (in)voluntary experiences, regarded as external and/or internal (and if internal, experienced as inner thoughts or speech), whether the sound is from own and/or (un)known voice(s), etc.). Also, large-scale cohort studies including young age ranges are still needed to unravel factors (biological, psychological, and social) that influence age of onset, persistence, and differential outcome.

To better understand the clinical relevance of hallucinations in children and adolescents and possible gaps in current health care, research assessment tools could structurally implement questions regarding the child’s need for care or current receipt of care, as research so far lacked to assess these aspects. Also, following findings of Kapur et al^101^ regarding difficulties when encountering mental health care for distressing hallucinations, it might be beneficial to also address clinician’s perspectives on their (un)certainties and needs when encountering distressing hallucinations, as this could contribute to the improvement of health care for children and adolescents. More uniformity in assessing the hallucinations should be strived for, whereas research could also focus on debating how it is possible that—giving the fact that they mostly ask more or less the same questions—that prevalence numbers vary so widely.^11^

## Summary

Despite the often transient nature of hallucinations in children and adolescents, these experiences, even at a young age: (1) can cause severe distress and reduced functioning; (2) can occur across diagnostic boundaries; (3) often go together with comorbid psychopathology; and (4) may cause or coincide with increased risk of suicidality. This profile is somewhat contrary to the working group’s proposition in 2014 to distinguish 2 types of hallucinations, those that occur in the presence of childhood psychosis and those that do not. Current insight suggests the application of a more general staging model, in which hallucinations can occur from a benign and transient phenomenon at one end to a symptom of severe psychopathology of several (interacting) domains at the other. Research since 2014 has not significantly advanced understandings of the etiology of hallucinations in youth, possibly attributable to: (1) the sparse amount of studies actually exploring the etiology and course of specifically hallucinations in youth (rather than UHR or psychosis’ first-episode samples), (2) the lack of data-driven narrowing of definitions regarding hallucinations, and (3) given that hallucinations can be related to (the development of) different psychiatric disorders, findings regarding etiology may be expected to be closely aligned to actual (underlying) psychiatric comorbidity. As a consequence, hallucinations in both child and adult studies still represent a broad phenotype. Nevertheless, although hallucinations in youth are often transient and possibly a benign phenomenon, children and adolescents seeking help for hallucinations often suffer prolonged from their hallucinations and encounter difficulties in receiving the appropriate care. Since 2014, clinical care has improved with the recent knowledge and development of youth-specific questionnaires and intervention strategies. Finally, although the implementation of large hallucination detection programs in the general population is unnecessary, further knowledge is required on the extent and (early) identification of children and adolescents with hallucinations that might be in need for care.

## Funding

C.F. is supported by a Wellcome Trust grant (WT108720). J.G.S. is supported by an Australian National Health and Medical Research Council Practitioner Fellowship grant (APP1105807). A.R. is supported by the Norwegian University of Science and Technology Onsager Fellowship Program in Psychopathology and Development (70440154).
